# Editorial Perspective: How spreading mental health information can be (un‐) helpful – a dynamic systems approach

**DOI:** 10.1111/jcpp.70055

**Published:** 2025-10-14

**Authors:** Daniele Marcotulli, Lucy Foulkes, Argyris Stringaris

**Affiliations:** ^1^ Department of Sciences of Public Health and Paediatrics University of Turin Turin Italy; ^2^ Department of Experimental Psychology University of Oxford Oxford UK; ^3^ Divisions of Psychiatry and Psychology and Language Science University College London London UK

**Keywords:** adolescence, anxiety, depression, diagnosis, over‐diagnosis

## Abstract

Increasing awareness of mental health problems, including that of young people, is generally seen as positive, and many interventions to increase awareness are underway internationally. Yet, a principled evaluation of the benefits and harms of increasing awareness is still lacking. Here, we present a conceptual framework for the evaluation of information interventions that are aimed at increasing public awareness of mental health problems. We borrow concepts from dynamic systems, such as infection spread and related population growth, to ask how benefits, but also harms of information on mental health, may accrue over time. We argue that as information spreads, several cascades of events are set off that involve members of the general public but also clinicians and healthcare services. These cascades entail positive and negative feedback loops. We discuss not only how increased diagnoses can lead to positive outcomes (e.g. increasing diagnostic rates and appropriate treatments in those who would otherwise have remained undiagnosed) but also how increased awareness can lead to decreases in diagnostic accuracy, to service overload, and how they may expose people to unnecessary or harmful treatments. We argue that the need for a framework founded on modelling societal dynamics is needed to ensure that both the benefits and the downsides of mental health information are accurately gauged and to help the planning of better public health campaigns.

## The problem

Advances in public health and medicine have meant that health outcomes and life expectancy have been improving steadily for several decades (World Health Organization, 2024a). Yet, mental health problems have been following the opposite trend, where – particularly in young people – problems such as depression and anxiety have been increasing. A major component of the burden of mental illness has long been seen as arising from associated stigma. Based on theory and empirical evidence (Henderson, Evans‐Lacko, & Thornicroft, 2013), the view has prevailed that increasing public awareness of mental health can reduce stigma and improve timely recognition and therefore treatment of mental health problems (Prince et al., 2007; World Health Organization, 2024b).

The underlying assumption, often implicit, of public health information interventions is that raising awareness will result in overall positive outcomes for the public; that is, that the benefits of efforts to increase awareness will outweigh harms (Wakefield, Loken, & Hornik, 2010) such as over‐diagnosis with resultant over‐treatment and potential iatrogenic harms (Foulkes, 2024; Foulkes & Stringaris, 2023) or diversion of healthcare resources. In this regard, the ‘Time to Change’ and ‘Every Mind Matters’ programmes have played a significant role in enhancing the public's understanding of how age, socioeconomic factors, pre‐existing cultural attitudes and communication‐related factors influence the impact of information interventions on the public (Walsh & Foster, 2021). However, when it comes to mental health problems, it is not clear whether raising awareness will always or most of the time result in net benefits (Hornik, Jacobsohn, Orwin, Piesse, & Kalton, 2008). Importantly, there is no current framework that allows for the principled evaluation of sources of harms and benefits of awareness efforts (Foulkes & Stringaris, 2023; Foulkes & Andrews, 2023; Foulkes, Andrews, Reardon, & Stringaris, 2024). This becomes increasingly important as potential adverse effects of not only pharmacotherapies but also large‐scale psychotherapy‐inspired interventions become more apparent (Guzman‐Holst, Streckfuss Davis, Andrews, & Foulkes, 2025; Klatte, Strauss, Flückiger, & Rosendahl, 2025).

In this paper, we argue that an approach founded on dynamic systems allows us to conceive of the richness and complexity of the effects that mental health information interventions can have on society. As such, this approach could prove invaluable in evaluating interventions and better planning future ones. We supplement our proposal with a formal account of this approach in the Appendix S1, including a software we have developed to illustrate the dynamic systems approach (*please note that the code used to illustrate the evolution of the dynamic systems has not been peer reviewed*).

## Overview: Re‐thinking societal mental health as a dynamic system

In this paper, we propose that every attempt to spread mental health awareness sets off a causal chain of events that involve as actors both the public and practitioners and that the outcomes of such a cascade are not guaranteed to be positive. Our proposal rests on previously developed ideas from population dynamics, and we draw analogies between the spread of mental health information and the spread of infections.

### Foundations in ecology, demography and infectious diseases

**Figure 1:**
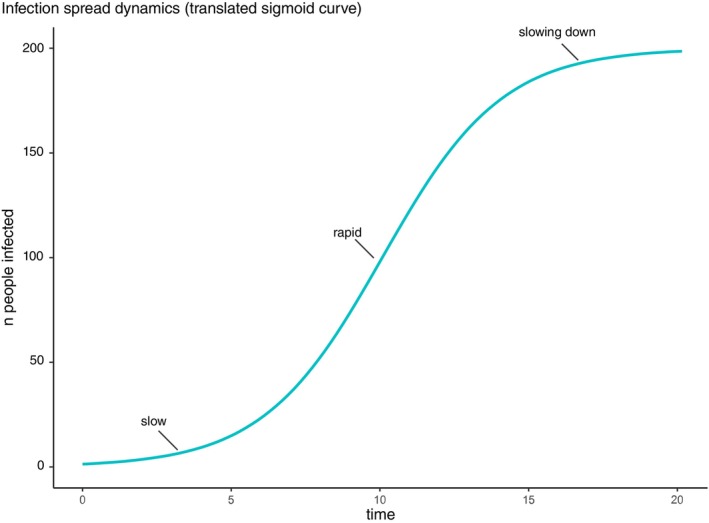
A graphical representation of spread in a population using infections as an example. The *y*‐axis represents the number of people infected in a population, and the *x*‐axis represents time. This sigmoid curve shows that the spread of the infection begins slowly, but as more people become infected (and can therefore facilitate further spread), the number of people infected grows steeply until it then plateaus, for example, because most people in the population have been infected. As explained in the text, infection is used as an analogy to information spread; also, not all spread is going to be sigmoid, though such types of spreads are quite common.

Indeed, while psychiatry and psychology are often concerned with the effects on individuals (as reflected in DSM and ICD nosologies), other scientific traditions are primarily concerned with populations and groups of individuals. Hence, demographers and ecologists may think of members of a society as parts of a *dynamic system*, that is a system that changes over time and whose change depends on initial and boundary conditions. For example, how a city population will grow over time will depend on how many people lived there at the beginning (initial condition) but also on the rate at which buildings can be erected to accommodate inhabitants (boundary condition). Similarly, how an infection will spread in a population will depend on factors such as the number of people who are immune or susceptible to the pathogen in question but also on what the density of the population is and how much people travel between locations. These dynamic systems often give rise to phenomena that are best described by curves that have sigmoid (S‐like) shapes. Imagine a curve with the number of infected people on the *y*‐axis and time on the *x*‐axis: infection spread may be slow initially as only a few people carry the germs (the curve will be flat), but as more become infected, the rate of the spread rises and the curve becomes steep, only for the curve to become flat again as soon as population level immunity has been achieved (Figure 1).

#### The concept of information spread

The need to think in such population terms in psychiatry and psychology should be obvious when it comes to interventions, such as awareness campaigns, that often target either the whole population or large swathes of it. Indeed, *information interventions*, such as mental health literacy campaigns (Hahn, Chua, Jones, & Henderson, 2023; Henderson & Thornicroft, 2009), convey to the general population information about the state of the world, e.g. how common it is to have depression, or that the symptoms of ADHD may be a brain‐based disorder. Therefore, the first step in applying this approach to mental health is to consider **information as something that has germ‐like properties** (we use this to describe the fact that people can “catch it” from others and *not* in a moral evaluative sense), i.e. has the capacity to spread amongst people, for good or for worse (Del Vicario et al., 2016; Meng et al., 2025; Zhou et al., 2020).

#### Members of society as actors–recipients in a dynamic system

The next step is to consider the initial conditions of the dynamic system, as described above. In order to do so, we need to think about two key components of the system. One is **obviously the general public**, **or in any case the part of the public targeted by the intervention**; members of the public are the recipients of the intervention, and they will vary in how *sensitive* they are to the information they are given. Some people may be prepared to listen to the intervention and agree; others may ignore it or choose to dismiss it. Importantly, as in a model of population growth or infection control, these recipients of information interventions are not passive; they can also **change their own behaviour**—in this case by help‐seeking, which is the intended outcome of many interventions (Da Conceição, Mesquita, & Gusmão, 2024; Xu et al., 2018). But they can also **change the behaviour of others**. For example, a person who learns something from a campaign may pass on the information to their friends or relatives (Alho et al., 2024). In that sense, recipients of information are also actors, and we therefore call them **actor‐recipients**.

#### Healthcare professionals as validators and controllers of help‐seeking

Among these actor‐recipients is a particular subset, namely **healthcare professionals**, such as psychiatrists, psychologists, nurses or social workers. They are recipients of information coming from the intervention, but they are also influenced by shifts in their patients' opinions and behaviors, and they may adjust their behavior accordingly. As an example, consider a general practitioner who, following a successful public awareness campaign, deals with many more referrals of people who are more informed about psychiatric conditions than people were before. It is also obvious that healthcare professionals are privileged *actors*: they act like members of the general public (e.g. information exchange), but they also have a major effect on individuals and the public as they are (a) ***validators of help‐seeking***, for example when they label a condition through a diagnosis; (b) ***controllers of treatment***, that is they decide about whether a person should or should not receive a treatment. These acting properties of **healthcare professionals mean that they are in a reciprocal (passive‐active) relationship with the effects of interventions, that is they are both affected by and they influence themselves the overall impact of the intervention**.

## Information intervention as a causal chain of events in the population

### The compartments of society

**Figure 2:**
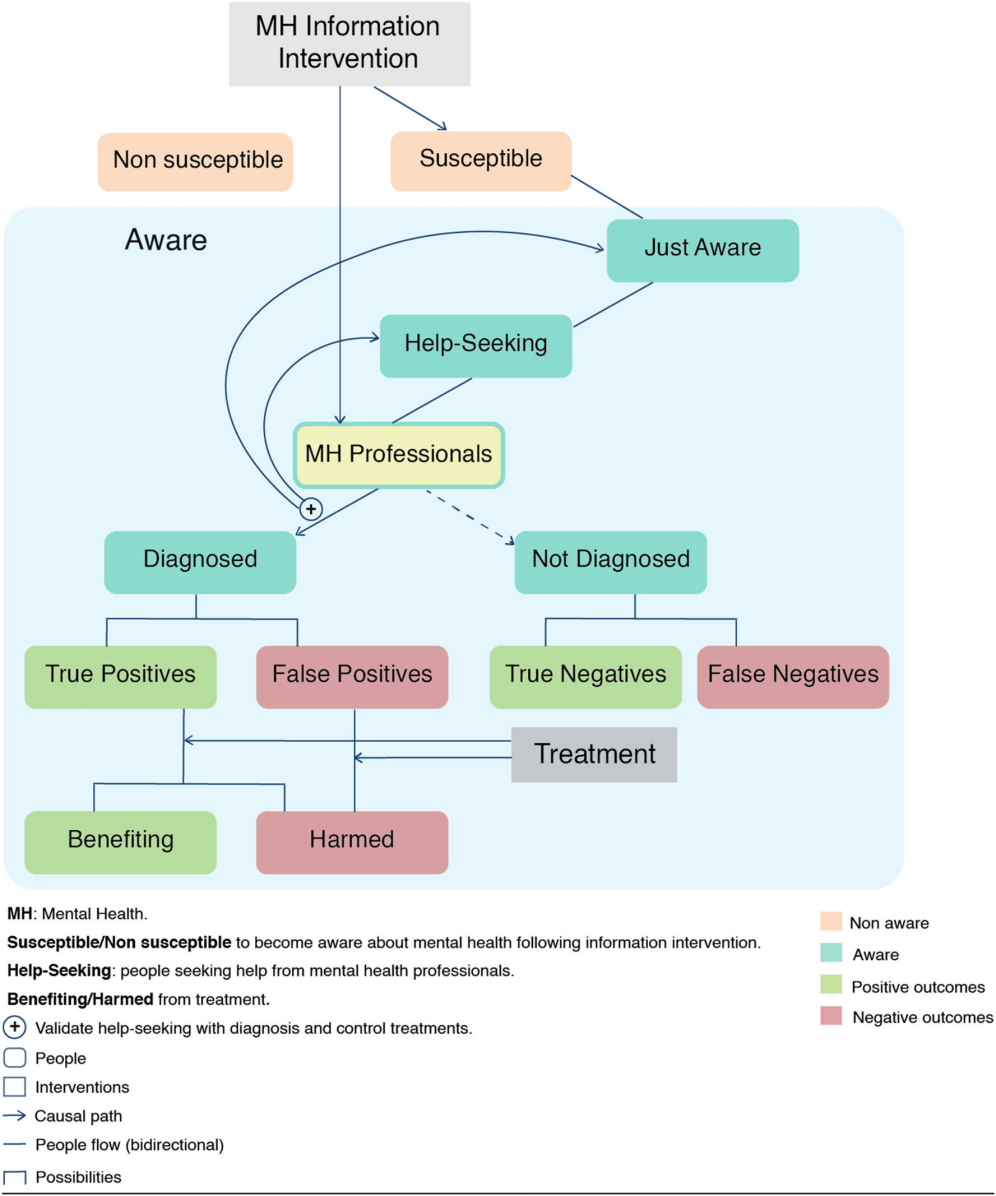
Framework dynamics. Schematics depicting the main dynamics modelled in the developed framework.

To depict the effects of an information intervention, it is useful to think of the members of a society as belonging to so‐called compartments, a technical term that describes the membership of people to groups according to some characteristics. In our case, we have three compartments, which, as we will see, have important effects on the spread of information:

(i) one compartment made up of **people who are already aware** of mental health problems – think of people with superior knowledge about mental health conditions; (ii) another comprising people **susceptible to becoming aware** of mental health problems – think of those who are keen to learn about mental health; (iii) one of **non‐susceptible individuals**, that is, those who for a variety of reasons are not affected by such campaigns (be it because the information does not arrive to them, e.g. social‐media‐delivered messages in elderly populations, or because they choose to ignore them). At any given time, a person can only belong to one of these groups, but can move from one group to the other over time. While we include non‐susceptible people in our model structure, we do not actively consider them throughout our modelling (Figure 2). This is because individuals in this group are hypothesised to be unaffected by the intervention.

### Effects of people aware on the spread of information

As we stated above, in any dynamical system, understanding the initial condition is important. Here, the compartments provide information on these initial conditions, and we need to think about how this will play out in the spread of information. It is plausible to think that the more people are already aware, or the more people are susceptible to information, the bigger the effect of the information will be (see Equation 1 in the Appendix S1).

### Epistemic bubble and snowballing effects

How information spreads also depends on what we call **boundary conditions** above. For example, it is obvious that information spread via schools will be minimal during the holidays and maximal when all pupils are at school (Eames, Tilston, & Edmunds, 2011). More generally, the more frequent and longer contacts people have (i.e. the denser the network), the higher the speed at which information spreads; also, contact with already aware people, with people seeking help or with people with a psychiatric diagnosis influences awareness. This **peer‐to‐peer reinforcement** accounts for close personal relationships and the ‘**epistemic bubble**’ and snowballing phenomena in social media. It is to be expected that people with a diagnosis have the greatest influence on awareness spread (Alho et al., 2024), followed by help‐seekers, with those simply aware having the least impact on spreading awareness. Nonetheless, we acknowledge that some mental health diagnoses (e.g. anxiety and depression) can facilitate higher spread rates, while others (e.g. psychosis) (Macdonald, Hayes, & Baglioni, 2000; Cohen & Sokolovsky, 1978) often involve more fragmented social networks. These details are easily accommodated in dynamic models. Those interested in possible mathematical formulations can look at Equations 2 and 3 in the Appendix S1.

## Information intervention as causal chain of events on healthcare practitioners and healthcare capacity

### Effects of information interventions on how clinicians diagnose

One of the advantages of viewing interventions through the lens of dynamic systems is that rates of change in one part of the causal chain can have effects on another and vice versa. Indeed, a reciprocal (non‐linear) relationship probably holds between the behaviour of the general public and practitioners. This is because it is reasonable to assume that practitioners adjust whether and how often they diagnose a condition in relation to changes in the social perception of a disorder. As more people become aware of a disorder/condition, more people will seek help for it, and not only **pressure on clinicians to diagnose increase but also clinicians' criteria may shift**. It is reasonable to assume that this happened with certain neurodevelopmental disorders in Britain (Mandell & Palmer, 2005; Hinshaw & Scheffler, 2014) and at least for a certain period of time with bipolar disorder in the USA. For example, clinicians may be more likely to diagnose people who until recently may have been labelled as subthreshold or who score highly on some but not all symptoms. This change in the diagnostic behavior of practitioners has two consequences: (i) it increases the prevalence of the diagnoses for any particular disorder directly—as the public and clinicians become more aware or clinicians come under pressure, they diagnose more of the disorder; (ii) the increased rate of diagnoses can itself have a reciprocal effect on the public: more people carry a diagnosis and this can itself act similarly to an awareness intervention and increase the number of people seeking help (and a diagnosis).

### Effects of information interventions on how accurately clinicians diagnose

**Figure 3:**
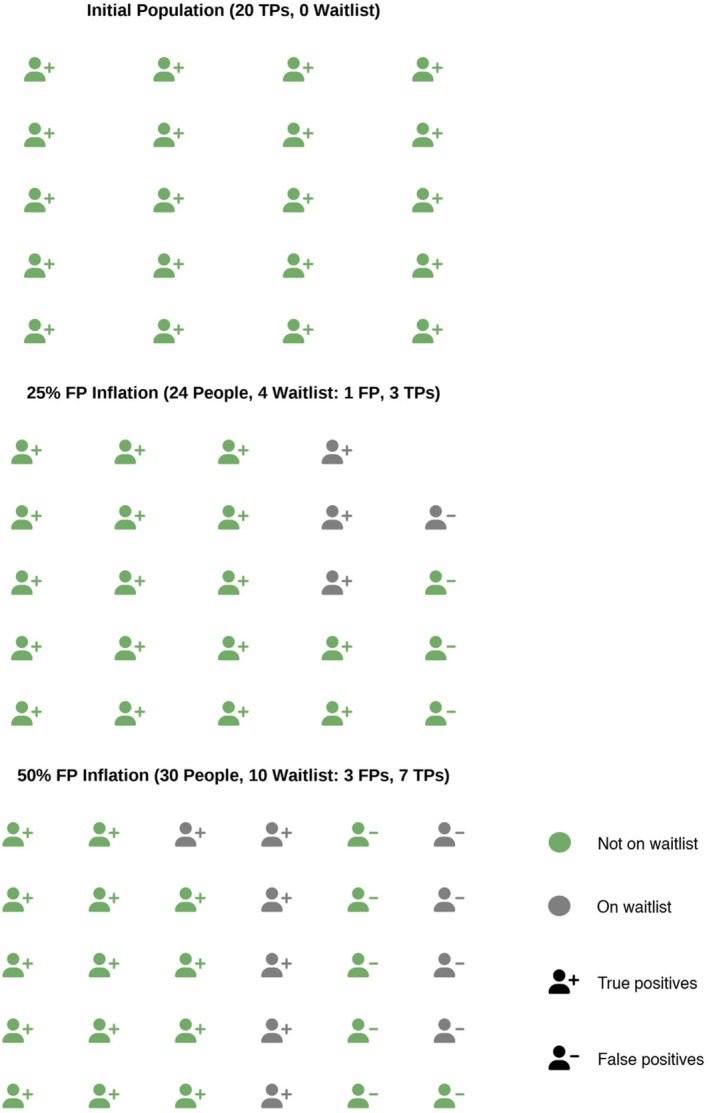
Effect of increasing help‐seekers and false positives rate on the health system capacity. As the number of individuals seeking help increases and therefore the prevalence decreases, the overall diagnostic accuracy may decline, resulting in a higher rate of false positives due to a reduced PPV. Because mental health systems have limited capacity, this surge in false positives can drain resources and lead to some people not receiving the support they need. In turn, true positives risk being delayed and never adequately treated, further compromising timely and effective treatment for those most in need.

It is often under‐appreciated that changes in how frequent a problem is in a sample (e.g. people who come to a clinic) will also change our accuracy in detecting it. Yet, it is a well‐known fact in epidemiology that the rarer a diagnosis, the higher is the rate of false positives, all else being equal (i.e. same clinicians, same diagnostic instruments). How does this apply here? A very likely consequence of increased awareness will be that people who do not carry a diagnosis (either the particular one or more generally) will become aware and may also become help‐seekers. This means that clinics will have a higher rate of people coming who should not receive a diagnosis. This creates a dilution effect: the proportion of people with a diagnosis will decline in clinic samples, and that will increase the rate of false positives. Figure 3 displays this effect, and please refer to Equations 4–7 in the Appendix S1 for the mathematical formulations.

This increase in false positive rates is yet another potential source of reciprocal effects: more people diagnosed means a higher probability of increasing awareness and help‐seeking in others, as with the changes in diagnostic behaviour. However, increases in false positive rates also have potential effects on the ability of healthcare providers to deliver treatments, but also the harm‐benefit ratio of treatment for mental health problems. We expand on these below.

### Effects of information interventions on healthcare providers' capacity

Because information interventions can lead to **an increase in the rate of false positives, this may affect the carrying capacity of a healthcare provider** (see Figure 3) – a larger proportion of people who don't have a true diagnosis will enter the system as diagnosed individuals seeking care, therefore potentially preventing others who truly have a diagnosis, from receiving services.

Also, because information interventions can lead to an expansion of the boundaries of the disorder and the lowering of the diagnostic threshold, the proportion of people with less severe disorders will increase. Even if the lowering of the threshold is generally justified, the consequence is that services and practitioners operating in a system of finite resources will have to struggle with prioritising cases. Also, as we explain below, treatments that apply to more severe cases may not apply to less severe cases, leading to problems with treatment choice and safety.

## Impact of information interventions on treatment outcomes

There are obvious benefits that may accrue from information interventions. As more people who truly have a condition (and would previously remain unrecognised) get diagnosed, more people in need will receive appropriate services. This important advantage needs to be balanced against the possibility of harm that presents itself due to changes in diagnostic practice and accuracy.

**Figure 4:**
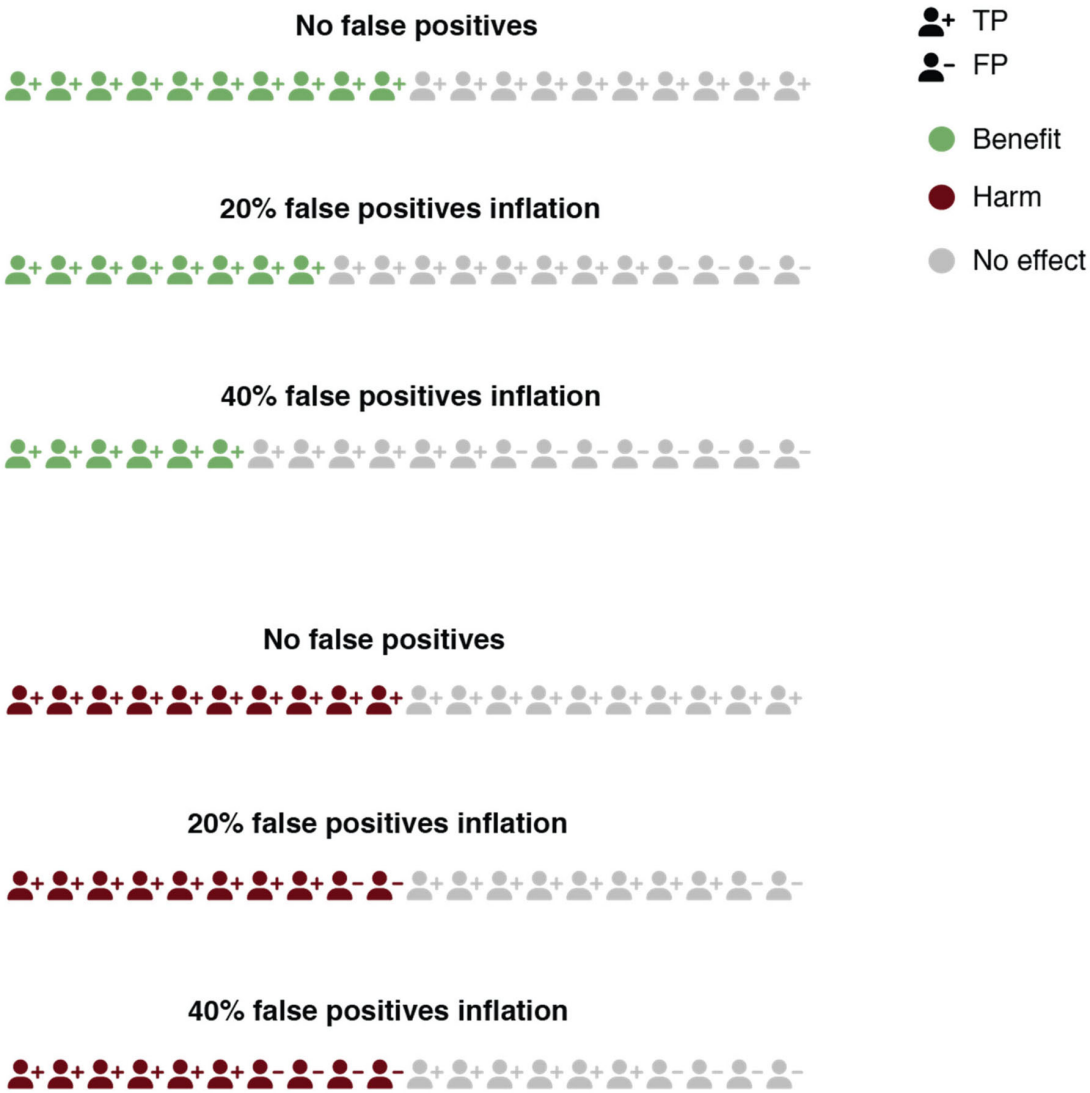
Effects of FP and TP on harms and benefits. If the total number of treated individuals remains the same, the number of those who are harmed does not change; however, when the proportion of false positives increases, fewer individuals genuinely benefit from the treatment

As discussed above, a likely consequence of information interventions is that people will be diagnosed who shouldn't have been diagnosed (false positives) and that people with milder forms of a disorder get increasingly diagnosed (lowering of diagnostic threshold). Both these shifts that arise may make the harm‐benefit ratio less favourable for the following two interrelated reasons: (i) the efficacy of an intervention may be higher in people with more severe disorder or may be unknown in those with less severe disorder (Kirsch et al., 2008; Stone et al., 2022); (ii) more people are exposed to the treatment and therefore its potential adverse effects; importantly, more people without a disorder (false positives) or people with less severe disorder will be put at risk of such adverse effects. We illustrate this in Figure 4 and provide a formal treatment of the problem using Numbers Needed to Treat in the Appendix S1.

Framing treatment effects as a balance between benefit and harm using the number needed to treat (NNT) versus the number needed to harm (NNH) is especially revealing, for example, for the expanding long‐term use of antipsychotics in minors with autism spectrum disorder (ASD) or bipolar disorder (Olfson, Blanco, Liu, Wang, & Correll, 2012; Radojčić et al., 2023). Short‐term trials show that risperidone alleviates irritability in roughly one child out of three (NNT ≈ 3). In the same interval, however, one in six gains at least 7% body weight and one in two experiences sedation (NNH ≈ 6 and 2, respectively) (McCracken et al., 2002). With continued treatment, these adverse effects accumulate, driving the effective NNH downward, while the behavioural benefit quickly plateaus. If the initial diagnosis is uncertain, the true NNT rises sharply, whereas the NNH for weight gain and sedation remains largely unchanged across conditions. At the population level, this combination (i.e. a higher real‐world NNT alongside a shrinking NNH) may shift the balance toward greater harm. Importantly, psychotherapies are not immune to similar issues, which are becoming increasingly concerning (Papaioannou et al., 2024; Foulkes et al., 2024).

Although psychosocial interventions have traditionally been viewed as relatively benign – largely under the assumption that, at worst, they might prove ineffective or impose unnecessary costs – emerging evidence indicates they can, in fact, carry significant risks of harm (Lorenc & Oliver, 2014; Foulkes & Stringaris, 2023). This risk may manifest in various ways, for example generating feelings of worry or guilt, exacerbating the existing targeted symptoms (Deighton et al., 2025) and driving an increase in false positives as detailed below.

Recent RCTs of school‐based information‐based interventions demonstrate mental health symptom deterioration in those receiving the information intervention. Indeed, findings from both the Youth Aware of Mental Health (YAM) program and The Mental Health and High School Curriculum Guide (The Guide) indicate that these interventions were associated with improvements in attitudes towards mental health as well as increased emotional difficulties (Deighton et al., 2025). For instance, for The Guide intervention, there was an increase in emotional difficulties (Standardised Mean Difference (SMD) = 0.09, 95% CI: 0.03–0.15) 1 year later among those receiving the intervention. Although this effect size is modest, its overall impact could be significant when applied to a large population. For instance, if 100,000 pupils receive the intervention, it is estimated that 1,500 pupils may experience relevant emotional difficulties due to the intervention, given that the NNH is around 65 (taking into account the psychometric properties of the assessment tool (Kwong, 2019) and the scaling of small effect sizes (Carey, Ridler, Ford, & Stringaris, 2023)).

As a result, it is increasingly recognised that psychosocial interventions should be assessed with the same level of rigour as pharmacological treatments, ensuring thorough harm–benefit evaluations and appropriate safeguards to minimise potential adverse outcomes (Foulkes et al., 2024; Papaioannou et al., 2024).

## Conclusions

In this paper, we present a conceptual framework (with reference to a mathematical foundation and its software application in the Appendix S1) to fill the gap in knowledge about how to assess the benefits and harms that may accrue through public health interventions that involve information.

We show that understanding the effects of information campaigns as influences within a dynamic system, in which various components may influence each other and change over time, is crucial.

Specifically, we argue that changes in awareness depend on pre‐existing (baseline) awareness and that increased awareness in its own right has knock‐on effects on help‐seeking and diagnosing. Similarly, the very process of diagnosing could be influenced by changes in the number of help seekers as well as by societal demands, thus leading to a decay of diagnostic accuracy. This in itself may change the probabilities of who gets the right diagnosis. The problem is probably particularly acute for a discipline such as psychiatry, where diagnoses rely solely on self‐report or clinical observation and lack biomarkers.

The ultimate question, then, is whether increased awareness and diagnosis yield more benefit than harm, a judgement that depends both on the time window of observation and the specific benefits of obtaining a diagnosis (Kazda, McGeechan, Bell, Thomas, & Barratt, 2022). While we often assume that having a diagnosis is inherently beneficial, this assumption may hold for some conditions but remains controversial for others (Levinovitz & Aftab, 2025).

By only focusing on the net benefits and harms experienced by individuals who receive treatment, our current modelling approach ignores broader, system‐wide consequences. For instance, increasing help‐seeking behaviours (potentially driven, at least in part, by false positives, as described above) can increase waitlists, exacerbate pressure on health systems, and ultimately broaden opportunity costs of help‐seeking itself. This dynamic may ultimately hinder some individuals with genuine clinical needs (true positives) from receiving help and further widen existing inequities. Expanding our scope to incorporate these systemic effects is essential for a more extensive and accurate assessment of public health intervention outcomes. The models we have developed serve as illustrations at present of the potential to predict harms and benefits within dynamic systems. The models' predictions should be tested against real‐world data, and the models calibrated accordingly to help policymakers and clinicians in decision‐making.

Clinicians, policymakers, and members of the public alike are faced daily with risk–benefit decisions. Often, such decisions are made without an explicit conceptual and mathematical framework and are intuitive rather than data‐driven. This can be particularly problematic for public health interventions, of which information campaigns for mental health are a prime example, where both benefits – but crucially also harm – scale. As with many public health interventions, for mental health information interventions, the potential exists for harm to outweigh any anticipated benefits, depending on specific parameters (many of which our framework can accommodate) and contexts. Importantly, when interventions are deployed on a large scale, any unintended harm can become substantial, leading to an extensive cumulative impact on the population, that is it can affect big populations of vulnerable people. Indeed, effects that are small at the individual level can have big impacts at the population level. To mitigate such risks, it is often advisable to start with smaller experimental or pilot studies and combine their insights with simulations or modelling across larger populations. By doing so, researchers and policymakers can more accurately gauge potential adverse effects, refine their intervention strategies, and ultimately minimise the likelihood of widespread harm.

## Ethical considerations

This does not apply as no human participants or animals were involved in the study.

## Supporting information


**Appendix S1.** Mathematical description of the framework and modelling details.

## Data Availability

Data sharing is not applicable to this article as no datasets were generated or analysed in this study.
