# Supplementary material for: Editorial Perspective: How spreading mental health information can be (un‐) helpful – a dynamic systems approach
Source: J Child Psychol Psychiatry. 2025 Oct 14;67(3):429–38. doi: 10.1111/jcpp.70055 (PMC12883590; doi:10.1111/jcpp.70055)
Supplement: Supplementary file 1 — Appendix S1. Mathematical description of the framework and modelling details. [file JCPP-67-429-s001.docx]

***How spreading mental health information can be (un-) helpful: a dynamic systems approach***

**Supporting Information**

**Appendix 1**

*Modelling of the direct effect of information intervention.*

We assume that the effect of *information intervention* increases with both the number of *susceptible* individuals and the proportion of aware people within the total population. These relationships can be mathematically expressed as:

*Equation 1:*

$$direct\_information\_effect=\left( A/N \right)S\alpha$$

Where:

- A is the number of already aware people.
- N is the total population.
- S is the number of susceptible people.
- α is a parameter reflecting the efficacy of information intervention and ranging from 0 to 1

In equation 1, the impact of an information intervention is set to be greater if the proportion of aware individuals is higher., until saturation. Similarly, the effect will be greater when the susceptible population is larger (the number of people that could potentially transition to the *aware* state is proportional to the number of susceptible individuals).

*Modelling of the indirect effect through individuals’ networks.*

We model the effect of the individuals’ network (i.e. the indirect effect of the information intervention) using a sigmoidal function derived from the standard population growth curves and basic infection spread models. We assume that the individuals’ networks effect increases with network density (i.e. how many and how frequent contacts individuals have). Similar to the direct effect, the indirect influences susceptible individuals (S) and its impact increases when there are more susceptible individuals (S).

*Equation 2:*

$$indirect\_information\_effect=\frac{max}{1+ e^{-\kappa\frac{(D*2) + (HS*1.5) + (A - HS - D)}{N}}} S\rho\lambda$$

- *max* is a carrying capacity, defining an upper limit to the effect.
- D is the number of diagnosed individuals (who are arbitrarily assigned a doubled weight in awareness spread)
- HS is the number of help-seeking individuals.
- To avoid double counting individuals, we subtract the number of diagnosed individuals and the number of individuals seeking help from the total number of aware individuals (A).
- We set the parameter ω to be 0.5 to simulate the requirement for at least two close contacts with individuals with a mental health condition before a subject starts seeking help (halved steepness). The parameter ω controls the steepness of the curve, with smaller ω reducing the rate of awareness increase. Figure 2.
- ρ is network density.
- λ is a parameter that controls the impact of the network on awareness spread.

To summarise, the total impact of an intervention is the sum of the direct and indirect effect. The number of aware individuals at any given time is modelled by subtracting the number of individuals losing awareness from those acquiring awareness due to the intervention:

*Equation 3:*

$awareness = direct + indirect information\_effect- \varepsilon A$

Where:

ε is the awareness fading rate.

Similarly, we model the process that leads aware individuals to seek help. The main difference from what is described above is that this process affects the already aware individuals.

*Modelling the diagnostic process*

To model the diagnostic process in the context of dynamically changing help-seeking behaviours, we take into account the limited capacity of the mental health system by defining a strict saturation parameter, which – we assume - saturates at 30% of the total population. Indeed, most mental health systems are taking care of less than 30% of the population and significant workforce shortages are observed (Health Resources and Services Administration).

The saturation parameter (σ) is defined as:

*Equation 4:*

$\sigma=1-\frac{\frac{D}{N}}{0.3}$

The probability that a practitioner diagnoses a psychiatric disorder (W_d_) is influenced by both the information intervention and societal pressure, which is proportional to the number of already diagnosed individuals. We assume that the probability of diagnosis W_d_ increases exponentially with the proportion of already diagnosed individuals (D/N), according to the following equation:

*Equation 5:*

$$W_{d} = 1 - e^{-\beta\frac{D}{N}}$$

Where:

β is a constant parameter that could be translated to the weight of the impact of societal pressure.

Thus, the rate new diagnoses (*W_d_*) in the help-seeking population will be given by:

*Equation 6:*

$R_{d} =\omega\sigma W_{d} HS$

Where:

- ω is a constant parameter.

*Modelling true and false positive*

*Approach 1.*

We estimate the true positives from the total number of diagnoses using a Bayesian framework that allows us to update estimates based on prior knowledge, in this case, trends in both the number of diagnoses and false positives. As per our hypothesis, we assume that the false positive influx is non-negative. Conservatively, we set the false positive influx to be around 0, modelling it with a normal distribution with mean of 0 and standard deviation of 0.1.

For the results presented here and in line with literature (Richardson et al., 2010), we assume the initial false positive rate to be around 20%. We also allow the diagnostic specificity to change as the number of false positives changes.

The specificity of a test can be defined as:

*Equation 7:*

$Specificity = 1 - False Positive rate$

At any given time, the true prevalence of a condition can then be derived using the Rogan-Gladen method (Rogan & Gladen, 1978), which we bound between 0 and 1 to avoid overestimation of true prevalence.

*Approach 2.*

In an alternative approach, we consider that the diagnostic properties are dynamic, adapting to contextual factors and societal changes and expectations. Accordingly, we model the diagnostic sensitivity and specificity in a Bayesian framework where they are allowed to change over time in response to shifts in the help-seeking population and apparent prevalence.

*The number needed to treat (NNT) and number needed to harm (NNH)*

For the purposes of this paper, we focus on benefits and harms resulting from treatments offered to diagnosed individuals by professionals. For these, there typically exist (or there ought to exist) the metrics of Numbers Needed to Treat (NNT), and Numbers Needed to Harm (NNH). The NNT and NNH are effect size measures, indicating the number of patients we need to treat to observe one additional responder compared to placebo (NNT) or harmed subject over placebo (NNH). These measures are simply calculated as the inverse of the absolute difference between the response (or harm) rates of an intervention group and its control. For example, if a drug leads to a response rate of 60% and placebo to a response rate of 40%, then the numbers NNT = 5, because 1/(0.6-0.4). This means that we need to treat 5 people to observe 1 responder that would not have been observed in the control condition. The equivalent reasoning applies to side effects (NNH).
